# Interactive Voice Response System: Data Considerations and Lessons Learned During a Rectal Microbicide Placebo Adherence Trial for Young Men Who Have Sex With Men

**DOI:** 10.2196/jmir.7682

**Published:** 2017-06-09

**Authors:** Jose Bauermeister, Rebecca Giguere, Cheng-Shiun Leu, Irma Febo, Ross Cranston, Kenneth Mayer, Alex Carballo-Diéguez

**Affiliations:** ^1^ Department of Family and Community Health School of Nursing University of Pennsylvania Philadelphia, PA United States; ^2^ HIV Center for Clinical and Behavioral Studies Columbia University and New York State Psychiatric Institute New York, NY United States; ^3^ Department of Biostatistics Mailman School of Public Health Columbia University New York, NY United States; ^4^ Gama Project Department of Pediatrics University of Puerto Rico Medical Campus San Juan Puerto Rico; ^5^ Division of Infectious Diseases School of Medicine University of Pittsburgh Pittsburgh, PA United States; ^6^ Fenway Institute Fenway Health Boston, MA United States

**Keywords:** user-computer interface, speech recognition software, HIV, survey methodology

## Abstract

**Background:**

Rectal microbicides, if proven effective, may aid in reducing human immunodeficiency virus (HIV) incidence; however, demonstration of efficacy and effectiveness is contingent on accurate measurement of product adherence. Delays in self-report, in particular, may affect the accuracy of behavioral data.

**Objective:**

The aim of this study was to capitalize on mobile phone use by young men who have sex with men (YMSM), and examine the use of an interactive voice response system (IVRS) by YMSM aged 18-30 years enrolled in a multisite, 12-week microbicide safety and acceptability trial.

**Methods:**

YMSM (N=95) enrolled across 3 sites (Boston, Pittsburgh, and San Juan) were asked to report their use of an applicator applied placebo rectal gel product during receptive anal intercourse (RAI) using the IVRS. IVRS was available in Spanish and English. After the 12-week trial, we examined whether IVRS problems were associated with YMSM’s sociodemographic characteristics (eg, age, race and ethnicity, and education), sexual behavior, or recruitment site. We used a multinomial logistic regression to compare YMSM who experienced no IVRS problems (n=40) with those who reported one IVRS problem (n=25) or two or more IVRS problems (n=30).

**Results:**

We recorded 1494 IVRS calls over 12 weeks. Over half of the participants (55/95; 58%) experienced challenges using the IVRS during the 12-week trial. YMSM reporting greater RAI occasions during the trial were more likely to experience one (odds ratio [OR]=1.08, 95% CI (1.02-1.14); *P* ≤.01) or more (OR=1.10, 95% CI (1.03-1.16); *P* ≤.001) IVRS challenges. Greater educational attainment was associated with multiple IVRS challenges (OR=7.08, 95% CI (1.6-31.6); *P* ≤.01). Participants in the Puerto Rico site were most likely to report multiple IVRS problems.

**Conclusions:**

Although IVRS was a useful data collection technology in our trial, several challenges experienced by English and Spanish speaking YMSM diminish its overall acceptability. We discuss strategies to optimize future development of IVRS data quality protocols based on lessons learned.

## Introduction

The interactive voice reporting system (IVRS) has gained popularity given its potential to collect time-stamped, prospective behavioral data, and to reduce participants’ recall bias during data collection [1-3] For example, participants can call into the system and report their data, rather than waiting until their next scheduled face-to-face visit with the research team [4,5]. IVRS has several additional methodological benefits to face-to-face approaches [1]. These benefits include making data collection accessible at any time and from any place, easing individuals’ ability to participate in research if they experience scheduling or transportation-related barriers, and having greater cost-savings than face-to-face approaches. IVRS can also reduce literacy concerns by allowing participants to hear the questions and respond verbally and/or using a numeric keypad, allowing for assessments across multiple languages, and deploying complex, tailored skip-patterns based on participants’ answers [3]. From a technological standpoint, IVRS also reduces compatibility issues as participants can use their phone of choice to report their behaviors and reduce data entry errors through automation [6].

Researchers have used IVRS to monitor behaviors over short, intensive periods (eg, multiple assessments a day over a week) and to follow participants for longer study periods. Within HIV prevention and care, researchers have noted that IVRS is an acceptable method for participants to share sensitive information and might encourage greater privacy than face-to-face methods [7-11]. In a study comparing Hispanic college students’ use of IVRS with a Self-Administered Questionnaire (SAQ) and a timeline follow-back (TLFB) over a 3-month interval, Schroeder and colleagues [9] found that participants’ sexual and substance use behaviors were underreported in the TLFB and over-reported in the SAQ when compared with the daily IVRS reporting. In their analyses, however, Schroeder and colleagues noted that IVRS use varied based on participants’ sociodemographic characteristics (eg, age and sexual orientation), with younger and sexual minority participants trending toward a greater likelihood of placing IVRS calls than older and heterosexual counterparts [9]. These findings suggest that participants’ characteristics might result in differential acceptability and use of IVRS.

Building on prior research examining the use of IVRS in HIV prevention studies, we examined the use of an IVRS to collect behavioral data from a sample of YMSM recruited to participate in a multisite rectal microbicide acceptability and adherence trial. Beyond overall use, however, we examined the prevalence of IVRS-related problems experienced by YMSM during the 12-week trial. We then examined whether YMSM’s sociodemographic characteristics (eg, age, sexual orientation, and educational attainment), sexual behaviors during the trial, and trial site (eg, Boston, Pittsburgh, and Puerto Rico) were associated with IVRS problems. We used our findings to discuss strategies to optimize future development of IVRS data quality protocols based on lessons learned in our trial.

## Methods

### Study Participants

Study data came from a larger project called Microbicide Safety and Acceptability in Young Men [12,13]. The study received institutional review board (IRB) approval from all participating institutions, and all participants signed informed consent. After screening (stage 1A), YMSM participated in a run-in period in which they were asked to apply a rectal placebo gel using a rectal-specific applicator (stage 1B), followed by a safety trial in which participants applied tenofovir 1% gel using a vaginal applicator for rectal delivery of the gel (stage 2). The study took place in 3 sites: Pittsburgh, PA; Boston, MA; and San Juan, PR. Study candidates were recruited from clinics, bars, clubs, newspaper advertisements, and social networks. Recruitment materials indicated that the investigators were looking for YMSM (aged 18-30 years) for a study about their sexual health and their feelings about rectally inserting a placebo gel resembling a microbicide gel currently under development before receptive anal intercourse (RAI). Full protocol description (Clinicaltrials.gov NCT01283360) is presented in detail elsewhere [12]. We focused our attention on stage 1B where the IVRS was used (December 2010 to October 2012).

Among participants who received medical clearance in stage 1A, we selected those fulfilling the more stringent eligibility criterion of having had condomless RAI within the prior 3 months to participate in stage 1B. This allowed us to focus on those with more recent potential risk and invite them to enroll in stage 1B. After undergoing an informed consent process, receiving risk reduction counseling and provision of condoms, and updating their medical history, participants received 20 rectal applicators filled with a placebo gel and instructions to insert the entire content of 1 applicator rectally within 90 min before each RAI episode. We used an applicator specifically designed for the delivery of a rectal microbicide [12] filled with hydroxyethylcellulose (HEC) gel. Hydroxyethylcellulose is also known as the “universal placebo” because of its use as placebo in most gel microbicide trials. Six weeks after their first stage 1B visit, participants returned for the mid-trial follow-up visit and were dispensed up to 20 additional applicators to ensure they had 20 on hand for the next 6 weeks. Six weeks after the mid-trial follow-up visit, participants returned for the final follow-up visit of stage 1B in which they completed a Web-based computer assisted self-interview (CASI) and semistructured interview that included questions on gel and applicator use. Participants received US $50 for each study visit (4 visits for stage 1A and 1B) and US $50 for a completed video teleconference interview. They also received US $1 per used applicator returned at visits 2 and 3.

### Interactive Voice Response System (IVRS)

Over the 12 weeks of stage 1B, participants were instructed to call an IVRS after each instance of RAI and/or applicator insertion, or at least once a week if they did not have RAI. Participants could respond by voice or use their keypad. The IVRS system was available in English or Spanish. During visit 2, participants generated a 4-digit password to identify themselves within the IVRS and were trained on how to enter data into the IVRS, including completing a mock call into the IVRS. At each call, participants were asked to report the number of times they had used the gel, whether they had RAI or inserted anything other than the gel, and whether they experienced any problems with the IVRS. Participants who did not call into the system at least once a week were contacted by the IVRS and reminded to log their behaviors. Furthermore, to encourage use of the IVRS as a data collection tool, we incentivized participants with US $1 per call with a maximum of US $30 and an additional US $10 bonus a month if they called at least once a week. In total, participants could earn up to US $60 in stage 1B for reporting their product use via IVRS.

IVRS data was downloaded into an excel (Microsoft) spreadsheet at the end of the trial. We examined the frequency with which participants reported a problem when entering their data within the IVRS system, as well as the number of call entries that were incomplete (eg, hung up call) or sequential (eg, participant called into IVRS and had to call back again to revise [eg, mistyping] or finalize their entry). For this analysis, we coded an IVRS problem as a call where participants reported experiencing an IVRS problem or when an IVRS call was incomplete (eg, hung up call) or duplicative (eg, participant called again to complete their IVRS entry). Participants who indicated that they had an IVRS problem since their last completed call were given the option to leave a voicemail indicating what went wrong. A member of the study team transcribed these voicemails in English or Spanish. Spanish comments were subsequently translated into English. We include examples from these transcriptions in the Results section to illustrate participants’ problems when using the IVRS.

### Participant Measures

Sociodemographic data were collected via a Web-based CASI. Demographic information included age, race or ethnicity (white, African American, Latino, mixed or other), sexual orientation, and highest educational attainment (1=8th grade or lower, 2=some high school, 3=high school or general educational development [GED], 4=partial college, 5=college graduate, 6=some graduate school, and 7=graduate school degree). Participants also indicated whether they were currently in school (0=no; 1=yes) and employed (0=no; 1=yes). Participants were also asked to report the number of receptive anal intercourse occasions in the prior 3 months. Participants were identified by their site’s location (1=Pittsburgh, PA; 2=Boston, MA; and 3=San Juan, PR).

### Data Analytic Strategy

After examining the descriptive statistics for our variables of interest, we used SPSS (version 23; IBM Corporation, New York) to test whether YMSM’s sociodemographic characteristics, sexual behavior, or site of recruitment were associated with the odds of experiencing IVRS problems during the 12-week trial. We used a multinomial logistic regression to compare the odds of experiencing a single or multiple IVRS problems during the 12-week trial. Due to limited observations in several categorical variables (eg, race or ethnicity and sexual orientation), we collapsed these indicators into dummy variables (eg, white: yes or no and gay-identified: yes or no) in our regression analyses.

## Results

### Sample Description

Study participants (N=95) had a mean age of 23 years. The racial or ethnic composition of the sample was predominantly Latino (46/95, 48%) and white (33/95, 36%), followed by a fewer number of African American (9/95, 10%) and mixed or other race (7/95, 6%) participants. Most participants (86/95, 91%) identified as gay. Most of the sample (84/95, 88%) reported having at least some college education (see Table 1).

**Table 1:** Sociodemographic characteristics of young men who have sex with men (YMSM; N=95).

Variable		n (%)
Age (years), mean (SD^a^)		23.2 (3.2)
**Race or ethnicity**			
	White or European American	33 (34.7)
	Black or African American	9 (9.5)
	Latino or Hispanic	46 (48.4)
	Mixed or other	7 (7.4)
**Highest educational attainment**			
	8th grade or lower	0 (0)
	Some high school	1 (1.1)
	High school graduate or GED^b^	10 (10.5)
	Some college	44 (46.3)
	College graduate	27 (28.4)
	Partial graduate school	2 (2.1)
	Graduate school degrees	11 (11.6)
	In school	47 (49.5)
	Currently employed	60 (63.2)
**Sexual orientation**			
	Gay	86 (90.5)
	Bisexual	9 (9.5)
**Recruitment site**			
	Pittsburgh	28 (29.5)
	Boston	26 (27.4)
	San Juan	41 (43.2)
Receptive anal intercourse occasions (before 3 months), mean (SD)			17.0 (15.5)

^a^SD: standard deviation.

^b^GED: general educational development.

### IVRS Calls

The IVRS recorded 1494 calls from 95 participants over 12 weeks. We flagged 162 calls (162/1494, 10.8%) reflecting an IVRS problem. The most common problems resulted from mistyping a numeric answer (74/95 participants, 46%), experiencing challenges entering their answers into the system (27/95 participants, 17%), being disconnected midway through the call and having to call back (58/95 participants, 36%), or the system being inaudible (3/95 participants, 2%). We include examples of these IVRS problems using participants’ voicemail transcriptions in Table 2. Overall, 40 YMSM (42%, 40/95) did not have any trouble using the IVRS during the 12-week trial, 25 YMSM (26%, 25/95) had one problematic event across the 12-week trial, and the remaining 30 participants (32%, 30/95) reported two or more problems across the 12-week period.

**Table 2:** Examples of participants’ comments regarding problems with the interactive voice response system (IVRS) during the trial.

Type of problem	Frequency n (%)	Exemplary quotes
Mistyping a number	74 (46)	“I mistakenly typed the wrong number; I pressed 1 when I should have pressed 2.” “I had to repeat questions several times because it wasn't getting the button presses.” “Had to call 5-6 times because every time I tried entering my User ID, it would say ‘I’m sorry’.” “(Problem with) entering my passcode.” “The automated entry froze.” “I mistakenly dialed that I didn’t use the gel during sex, but I did use it.”
Being disconnected midway through the call	58 (36)	“I’ve called twice already and the call drops”. “Listen, I’ve tried to call 4 times and every time that I select an option, it indicates that I have nothing to say and hangs up on me. I’ve called 4 consecutive times. This time I pressed 1 to describe the situation.” “Had to call back.” “I was trying to record a message at the last time I called in and it stopped the message recording before I could say anything and it's happened more than once and I forget what I want to say the next time I call in because it doesn't let me re-record or anything.”
Challenges reporting their answers into the system	27 (17)	“It didn’t let me log into the system. Four days later it called me to indicate that I hadn’t called in for that week”. “The only problem with the system is that it kinda takes a bit long to go through it and you have to wait for each question to finish makes it difficult to use after you call you know what buttons to press and basically you have to wait and the call takes longer.” “I did not say why I did not use the gel it was probably because my roommate was in the room and I did not want to give a voice command but it was because I didn't have sex that I didn't use the gel.” “When I gave my answer to several questions, it skipped and said: ‘I’m sorry, the answer is invalid’”. “If there is any background noise while the phone system is waiting for me to press 1 or 2 it would say “I'm sorry that was not a valid-I'm sorry that was not a valid-I'm sorry that was not a valid” and if it was completely quiet then it would stop doing that and I was able to press the buttons.”
Inaudible system	3 (2)	“Your voice is not very clear at all, it's kinda blurry at some points.” “It was just cutting in and out.”

### Multivariable Regression

Using multinomial logistic regression (see Table 3), we examined whether IVRS problems were associated with participants’ sociodemographic characteristics, sexual activity, or study site. Compared with YMSM who did not experience any IVRS problems, YMSM who experienced one IVRS problem during the trial were more likely to have a greater number of RAI occasions during the 12-week trial. No other differences were observed between YMSM who experienced a single IVRS problem and those who had no IVRS problems.

YMSM who experienced two or more IVRS problems were more likely than YMSM without IVRS problems to report a greater number of RAI occasions during the 12-week trial, to report greater educational attainment, and to be currently in school. Participants in Puerto Rico were more likely to report two or more IVRS problems than peers in the Boston site. There were no differences between Boston and Pittsburgh. No other differences were observed between YMSM who experienced multiple IVRS problem and those with no IVRS problems.

**Table 3:** Multinomial logistic regression of interactive voice response system (IVRS) problems over a 12-week period (N=95). Racial ethnic minorities serve as race or ethnicity referent group. Bisexual men serve as referent group for sexual orientation. Puerto Rico serves as referent group for recruitment sites.

Characteristics	1 IVRS problem during 12-week period (N=25)			2+ IVRS problems during 12-week period (N=30)		
	OR	95% CI	Significance	OR	95% CI	Significance
Intercept			.33			.04
Age	1.03	0.83-1.29	.77	1.04	0.81-1.32	.77
White	2.59	0.48-14.06	.27	4.69	0.48-45.29	.18
Educational attainment	1.31	0.64-2.65	.46	2.48	1.15-5.35	.02
In School	1.96	0.52-7.39	.32	7.08	1.59-31.60	.01
Is employed	0.92	0.23-3.72	.90	0.78	0.17-3.54	.75
Gay-identified	0.53	0.08-3.36	.50	0.52	0.05-5.33	.58
RAI occasions (3 months)	1.08	1.02-1.14	.01	1.10	1.03-1.16	.001
Pittsburgh	0.48	0.07-3.22	.45	0.09	0.01-1.25	.07
Boston	0.20	0.03-1.32	.09	0.05	0.01-0.55	.01
−2LL=144.33; X^2^_18_=42.5, *P*<.001		Nagelkerke Pseudo R^2^=44.0%				

## Discussion

### Principal Findings

Technological advances continue to increase researchers’ ability to capture behavioral data in experimental trials and observational studies. These data collection technologies (eg, IVRS, SMS text messages [short message service, SMS], and activity trackers) may be deployed to monitor participants’ behaviors and contexts outside of the clinic setting and have the potential to be synchronized with other systems (eg, electronic medical records and e-based applications that support behavior change) [1]. In this study, we examined the use of an IVRS as a data collection tool in a rectal placebo gel acceptability and adherence trial with English and Spanish speaking YMSM. The IVRS recorded nearly 1500 calls over a 12-week trial period, highlighting its overall acceptability and feasibility among YMSM in HIV prevention studies.

Similar to other data collection methods, the use of IVRS as a data collection tool had its challenges. Ten percent of IVRS entries recorded during the trial were classified as having errors due to user (eg, user entering the wrong number to indicate their answer), system (eg, the IVRS not recognizing users’ voice responses when there is background noise), or connectivity (eg, bad cellphone signal causing a dropped call) issues. As such, researchers employing IVRS as a data collection tool should ensure that time and attention is placed on data quality assurances before protocol implementation and during data cleaning. Furthermore, most of the IVRS challenges observed in our study might decrease over time as new innovations emerge. Improvements in wireless infrastructure (eg, better signal strength across the globe), capabilities and programing of IVRS (eg, better voice-response accuracy and automated data cleaning clarification questions), and devices (eg, mobile phone features) may alleviate participants’ challenges when using an IVRS. For example, advances in IVRS programing could allow users to continue where they left off if they experience a problem (eg, call failure) and reduce frustrations stemming from having to restart their entry from the beginning. In situations when participants might feel uncomfortable verbalizing their answers (eg, someone walks into a room), designing opportunities for users to toggle their reporting through a multimodal response system (eg, switch from voice to text without interruption) may also improve IVRS data collection in real time.

Participants who reported greater RAI occasions during the trial were also more likely to experience IVRS challenges. Given that YMSM were instructed to call the IVRS every time they had RAI over the 12-week period, it is not surprising that those who contributed to a greater number of calls would have a greater likelihood of experiencing IVRS problems. After adjusting for participants’ sexual activity, however, we noted that the probability of experiencing one or more IVRS challenges differed across participants’ educational attainment and study site. YMSM with greater educational attainment reported more IVRS problems. These educational differences may be attributable to greater assertiveness to note problems with the system, and/or may be a proxy for socioeconomic differences regarding type of device (eg, mobile vs smart phone) owned. They may also have higher expectations about how efficient systems should work (they have more money, use high end devices, are more critical, and know what they can expect). In addition, participants in Puerto Rico were most likely to report having multiple IVRS challenges than peers in Boston. Although the IVRS had a Spanish version, it is possible that differences in participants’ speed and enunciation when speaking Spanish made it harder for the system to capture their data accurately. Compared with the mainland United States, optimal cell phone signal and connectivity in Puerto Rico is lower—particularly in the more rural areas of the island. Taken together, our results support Schroeder and colleagues’ findings [9] that participant sociodemographic characteristics may affect their use of the IVRS as a data collection tool.

### Limitations

Our study has several limitations that deserve a mention. First, we did not ascertain participants’ cellular plans or type of mobile phone during the study. Future research should consider how technological aspects (eg, type of phone and signal reliability) may affect data collection before IVRS implementation. In areas where signal strength or connectivity are a challenge, for instance, it may be better to rely on other methods (eg, an app notification system) that collects the information and stores it in the device in real time and subsequently transfers them to a central server once a connection is secured may be warranted. Third, our study focused on a sample of young men (ages 18-30 years) in 3 regions of the United States, limiting our ability to ascertain whether IVRS problems are similar or heightened in other age groups or contexts. Finally, our study took place from December 2010 to October 2012. The quality of IVRS is likely to have improved given the fast-pace of telecommunication innovations in society. Nevertheless, our study points to key data collection issues that must be considered as researchers plan and implement studies that rely on innovative data collection systems.

Overall, our study findings support the notion that IVRS is a feasible and acceptable method to collect time-stamped, prospective behavioral data from YMSM. As with other data collection methods, we encourage researchers to devote time and attention to the adequacy of IVRS for their populations of interest. Data quality assurances before protocol implementation, including considering how varying connectivity may create data collection challenges, are warranted. Nevertheless, even though some individuals might experience challenges, the interest and perseverance of YMSM to use this technology forecasts interesting possibilities that need to be explored in future research.

## Abbreviations

GED: general educational developement
HEC: hydroxyethylcellulose
IRB: institutional review board
IVRS: interactive voice recognition system
RAI: receptive anal intercourse
SAQ: Self-Administered Questionnaire
TLFB: timeline follow-back
YMSM: young men who have sex with men
